# Chikungunya virus in Asia – Pacific: a systematic review

**DOI:** 10.1080/22221751.2018.1559708

**Published:** 2019-01-16

**Authors:** B. M. C. Randika Wimalasiri-Yapa, Liesel Stassen, Xiaodong Huang, Louise M. Hafner, Wenbiao Hu, Gregor J. Devine, Laith Yakob, Cassie C. Jansen, Helen M. Faddy, Elvina Viennet, Francesca D. Frentiu

**Affiliations:** a Institute of Health and Biomedical Innovation, School of Biomedical Sciences, Queensland University of Technology, Brisbane, QLD, Australia; b Department of Medical Laboratory Sciences, Faculty of Health Sciences, The Open University of Sri Lanka, Colombo, Sri Lanka; c Institute of Health and Biomedical Innovation, School of Public Health and Social Work, Queensland University of Technology, Brisbane, QLD, Australia; d Mosquito Control Laboratory, QIMR Berghofer Medical Research Institute, Brisbane, QLD, Australia; e Department of Disease Control, Faculty of Infectious & Tropical Diseases, The London School of Hygiene & Tropical Medicine, London, UK; f Communicable Diseases Branch, Department of Health, Queensland Government, Herston, QLD, Australia; g Research and Development, Australian Red Cross Blood Service, Brisbane, QLD, Australia

**Keywords:** Arbovirus, mosquito, emerging virus, alphavirus, viral arthritis

## Abstract

Chikungunya virus (CHIKV) is a mosquito-borne pathogen that causes an acute febrile syndrome and severe, debilitating rheumatic disorders in humans that may persist for months. CHIKV’s presence in Asia dates from at least 1954, but its epidemiological profile in the region remains poorly understood. We systematically reviewed CHIKV emergence, epidemiology, clinical features, atypical manifestations and distribution of virus genotypes, in 47 countries from South East Asia (SEA) and the Western Pacific Region (WPR) during the period 1954–2017. Following the Cochrane Collaboration guidelines, Pubmed and Scopus databases, surveillance reports available in the World Health Organisation (WHO) and government websites were systematically reviewed. Of the 3504 records identified, 461 were retained for data extraction. Although CHIKV has been circulating in Asia almost continuously since the 1950s, it has significantly expanded its geographic reach in the region from 2005 onwards. Most reports identified in the review originated from India. Although all ages and both sexes can be affected, younger children and the elderly are more prone to severe and occasionally fatal forms of the disease, with child fatalities recorded since 1963 from India. The most frequent clinical features identified were arthralgia, rash, fever and headache. Both the Asian and East-Central-South African (ECSA) genotypes circulate in SEA and WPR, with ECSA genotype now predominant. Our findings indicate a substantial but poorly documented burden of CHIKV infection in the Asia-Pacific region. An evidence-based consensus on typical clinical features of chikungunya could aid in enhanced diagnosis and improved surveillance of the disease.

## Introduction

Chikungunya, a re-emerging tropical disease with a widespread geographical distribution [1], is caused by chikungunya virus (CHIKV), an alphavirus of the family *Togaviridae.* The virus is transmitted between humans through the bite of an infected *Aedes* mosquito, primarily *Ae. aegypti* or *Ae. albopictus.* Chikungunya is generally self-limiting, with patients experiencing high fevers, headache, nausea/vomiting, persistent and sometimes debilitating myalgia/arthralgia, and maculopapular rash [2,3]. It can lead to prolonged arthralgia, lasting for several months and severely reduce the quality of life in patients [4]. Although chikungunya is rarely fatal, the virus can be a significant cause of central nervous system disease in the context of a large outbreak, placing younger children and elderly with co-morbidities at risk [5,6].

Three major CHIKV genotypes have been identified: West African, East/Central/South African (ECSA) and Asian [7]. Since CHIKV’s first description in 1952 in Tanzania [3], intermittent outbreaks have been recorded in Africa, Asia, and the Indian Ocean Islands [8–10]. In 2004, a major epidemic started in eastern Kenya [11], spread to several Indian Ocean Islands, India, and South East Asia (SEA) [12,13] and severely affected La Reunion (2005–2006) [14]. The epidemic saw the development of clinical complications not previously associated with CHIKV [14], and adaptation of an ECSA strain (the Indian Ocean Lineage, or IOL) to *Ae. albopictus* vectors, resulting in enhanced transmission rates [15,16]. The adaptation has possibly facilitated worldwide epidemics and outbreaks over the past decade in *Asia*, the Indian subcontinent and *Europe* [17–19]. In 2013, CHIKV emerged in the Western Hemisphere, and since then, 46 countries from the Americas have reported local transmission, with over 1.7 million suspected cases [20]. CHIKV continues to cause outbreaks globally [21], and it is estimated that 39% of the world’s population lives in areas endemic for the virus [22]. Infections can result in significant morbidity due to the acute and chronic disability associated with the disease [23], placing a heavy burden on health systems and infrastructure, and resulting in significant socio-economic consequences to individuals [24,25]. Despite the large burden of disease and the almost continuous circulation of CHIKV in Asia since the early 1950s, the epidemiological profile of the virus remains poorly characterized for SEA and the Western Pacific Region (WPR). We systematically reviewed available records documenting CHIKV circulation in the countries of SEA and the WPR over the past 70 years, with emphasis on epidemiological history, clinical features, and distribution of virus genotypes. This knowledge will aid the development of strategies to detect, prevent and respond to future outbreaks, identifying at-risk populations, enhancing accurate diagnosis, and improving treatment efforts.

## Results

**Figure 1.**
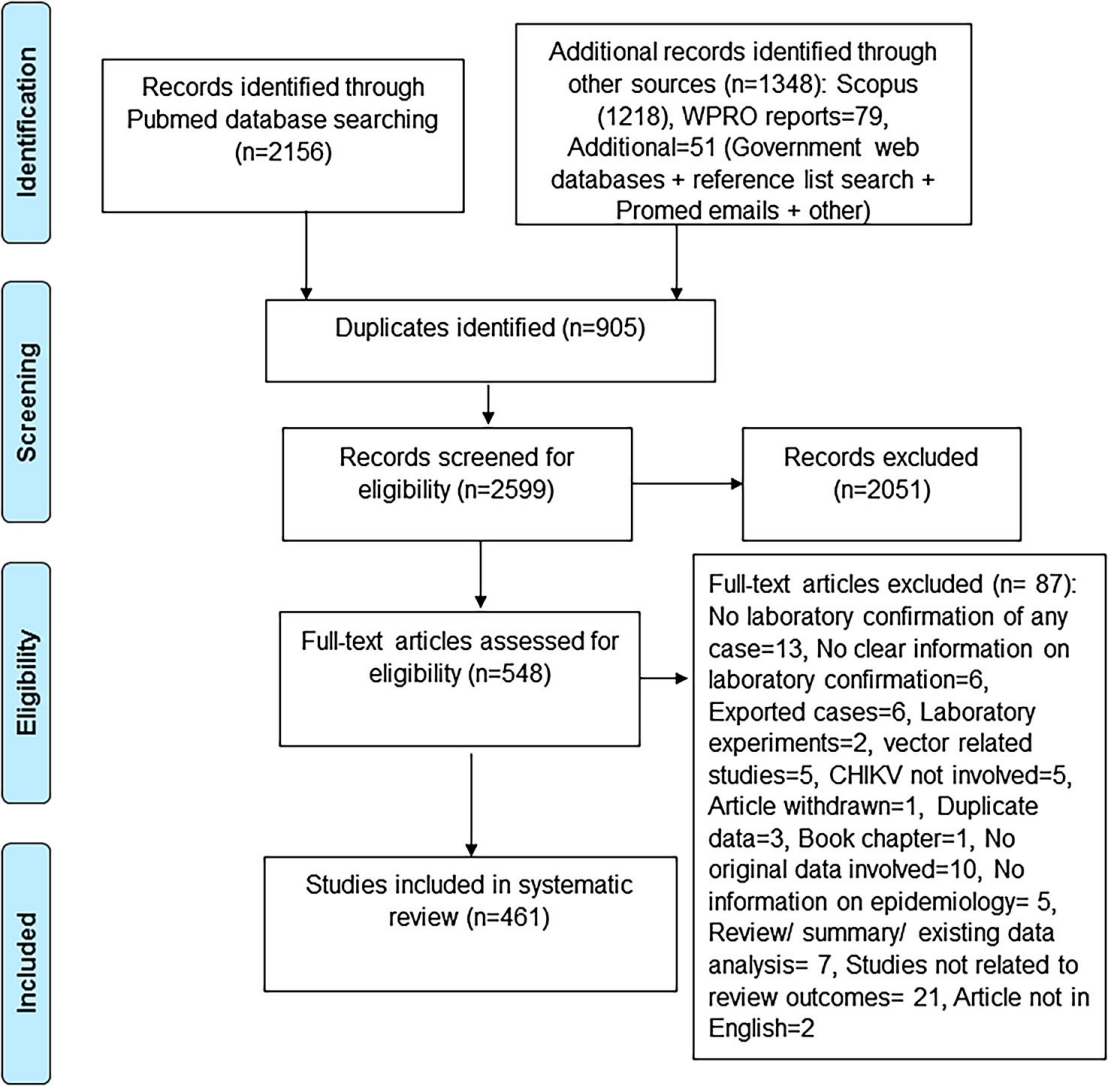
Flow chart of record selection for inclusion in the systematic review.

The selection process initially identified a total of 3504 records, from which 905 duplicates were removed (Figure 1). Among the remaining 2599 records, 2051 were excluded during the screening of abstracts as they did not meet the eligibility criteria. The full texts of the remaining 548 records were then assessed for eligibility. Following full-text review, 87 records were excluded according to criteria given in Figure 1. Therefore, a total of 461 records were considered for further analysis (see appendix pp. 1–15, Supplementary Table 1). Within these 461 records, 79 WPR syndromic surveillance reports were considered only for descriptions of place and time, but not for descriptions of other outcomes as many did not clearly describe laboratory confirmation of the cases.

### Epidemiology and chronology of CHIKV

**Figure 2.**
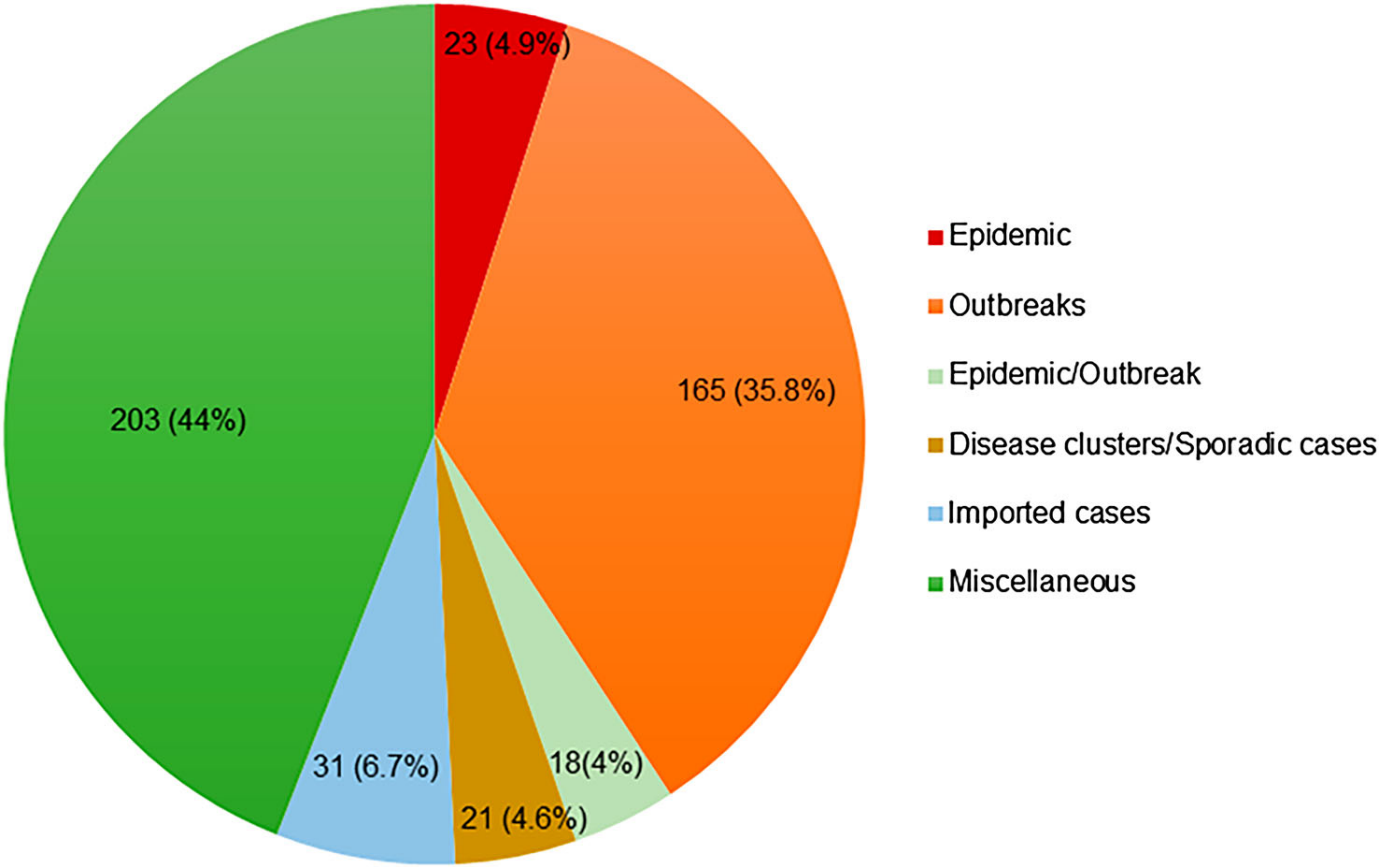
Distribution and number of records according to the type of CHIKV activity. The miscellaneous category includes surveillance/serological surveys/seroprevalence studies, atypical manifestations and other records that cannot be classified into any of the other categories.

Records were classified according to the type of CHIKV event: 23 records described epidemics, 165 described outbreaks, 18 had inconclusive categorization as either epidemic or outbreak, 31 reported imported cases, 9 reported disease clusters/case series, and 12 reported sporadic cases (Figure 2). A final category of 203 miscellaneous records included 149 surveillance/serological surveys/seroprevalence studies, 47 records on atypical manifestations, and 7 other records that could not be classified into any of the above categories. The person, place, and time of these records are described below.

### Person

There were 52 records describing the age and sex of persons affected by CHIKV (see appendix pp 1–15, Supplementary Table 1). Twelve records indicated that CHIKV solely affected adults and four records presented evidence of infection in both adults and adolescents. However, most studies (36/52) indicated that all age groups were affected. Among these, the highest number (11/36) indicated that patients 20–40 years old were the most affected, followed by people 40–60 years old (8/36). Only one study indicated that children aged 4–5 years old (reference 50, see appendix pp 41 for References) were most frequently affected, whereas two records indicated that people older than 60 years of age were affected. The latter two records described outbreaks in Maharashtra, India from January to December 2006, and in Guangdong province of China from September to October 2010. A further two records of an outbreak (Karnataka, India) at two different times (January to Sept 2008 and June 2008 to May 2009) indicated that people over 45 years of age were affected most frequently. However, a third of records (12/36) reported variable ages for persons affected by CHIKV. Almost 35% of records indicated that males were affected more than females, while another 32% indicated female predominance. A similar proportion (30%) of records indicated that both sexes were equally affected, with another 3% presenting no information. Overall, the evidence indicated both sexes suffer equal burdens of the disease.

### Place of occurrence

**Figure 3.**
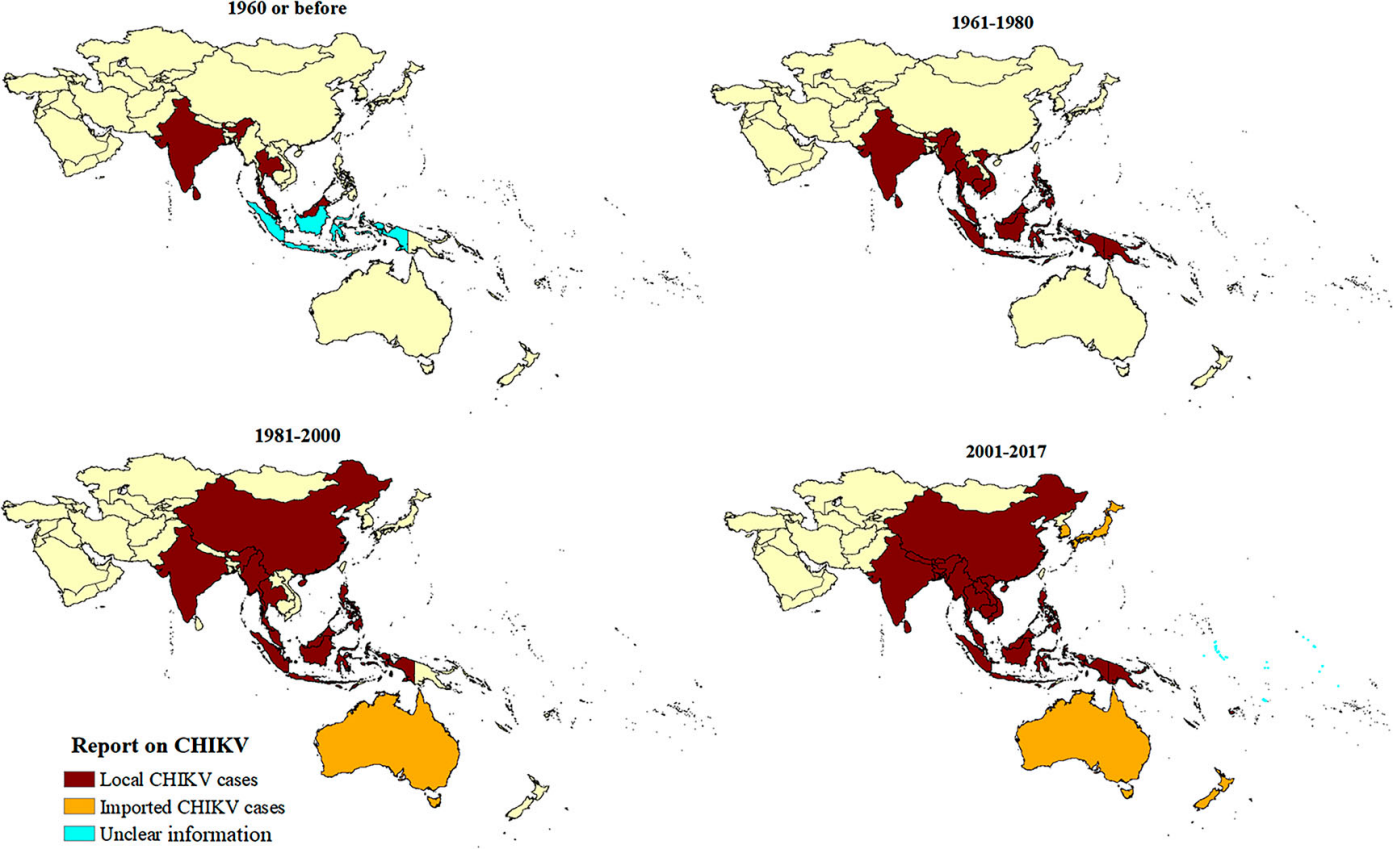
Spatial-temporal expansion of CHIKV in SEA and the WPR regions, including records of both local and imported cases.

The records indicated that CHIKV has steadily expanded its range in the Asia-Pacific over the last 70 years (Figure 3). India (174 records, 46%), Thailand (35 records, 9%), and Malaysia (26 records, 6.81%) have reported CHIKV circulation since the virus was first reported to have reached SEA prior to 1960 (see appendix pp 16, Supplementary Table 2) [26]. The SEA region accounted for 67.02% of records, and CHIKV epidemics were reported only from India, Thailand, and Sri Lanka. The WPR syndromic surveillance records were considered separately from the above, as they report multiple countries in the same report. American Samoa, Cook Islands, Marshall Islands, Kiribati, Nauru, Samoa, Tokelau, Tonga, and Tuvalu also reported CHIKV activity. However, information on laboratory confirmation of CHIKV cases was only available for some outbreaks. Only imported CHIKV cases were reported from Niue, Hong Kong, Korea, Japan, Cook Islands, New Zealand, and Australia.

### Time of occurrence

**Figure 4.**
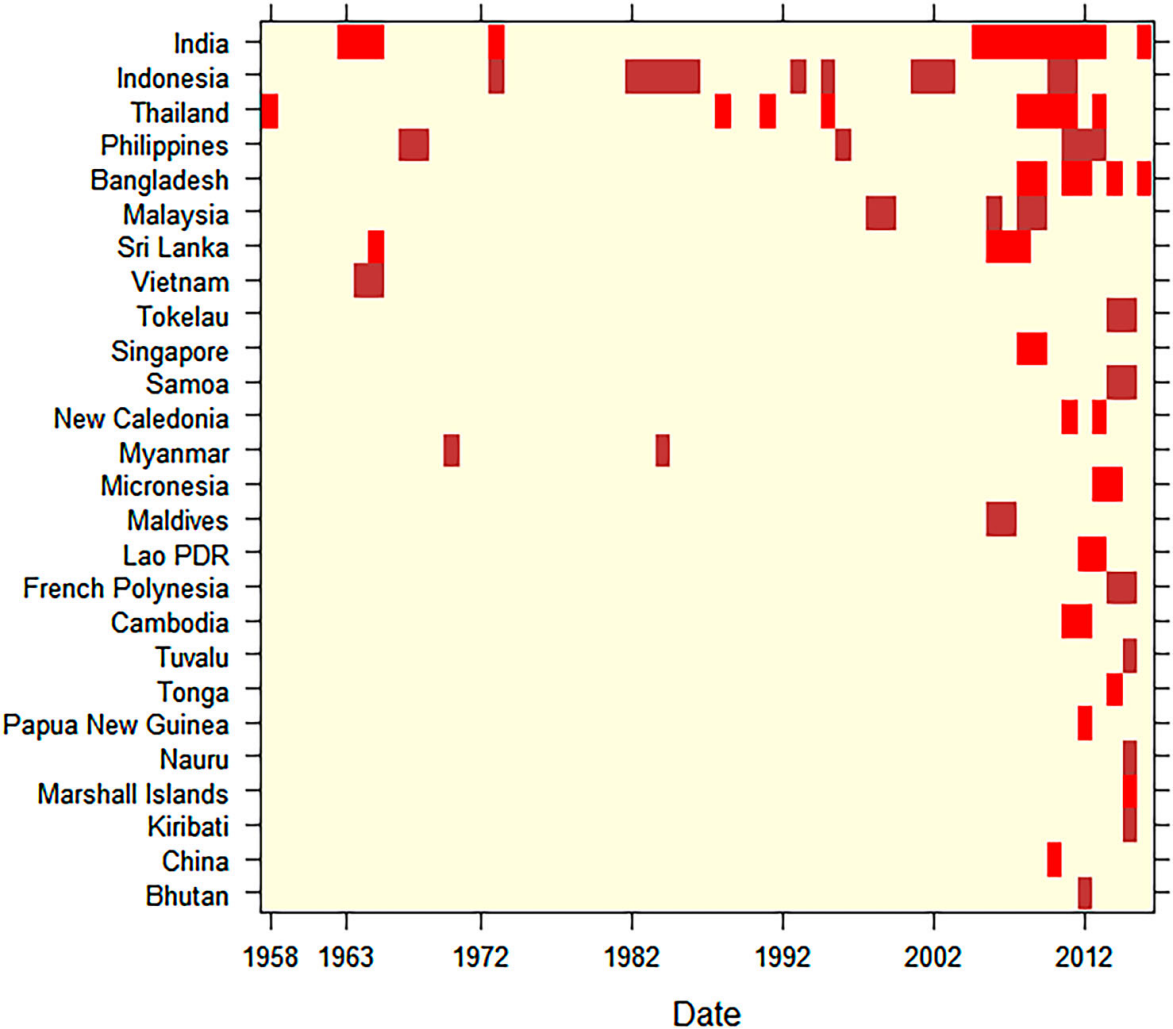
Heat map of CHIKV outbreaks and epidemics in the countries of SEA and the WPR regions, by year (1963-2017).

Prior to 2000, most records dated from the period 1961–1970. The two decades following the year 2000 accounted for nearly 83% of records identified (see appendix pp 17, Supplementary Table 3). From the 1950s to 2017, CHIKV outbreaks and/or epidemics most frequently occurred in India, Indonesia, Thailand, and Philippines (Figure 4). A retrospective analysis of human sera indicated that CHIKV was circulating in the SEA region in 1954. The first epidemic co-infection of CHIKV with dengue virus was detected in Thailand in 1958. India reported its first CHIKV outbreak in 1963. However, a disease with similar clinical features was first noticed in 1779 from Indonesia, although a number of infectious agents can lead to similar symptoms [27]. Among WPR countries, Singapore reported the first CHIKV activity in 1960 while Cambodia reported the first disease cluster infected with CHIKV in 1961. Within the Western Pacific islands, New Caledonia reported its first outbreak in 2011. Intervals between CHIKV outbreaks were observed in Sri Lanka (41 years), India (32 years), Philippines (28 years: 1968–1996, 15 years: 1996–2011), Myanmar (14 years), Thailand (13 years), Malaysia (7 years), and Indonesia (6–8 years) (see appendix pp 17 for Supplementary Table 3). The majority of countries in SEA and the WPR have experienced CHIKV outbreaks and/or epidemics only since the mid-2000s (Figure 4).

### Attack rate

The attack rate during specific CHIKV outbreaks and epidemics was described in 22 records (see appendix pp 18-19, Supplementary Table 4). The clinical attack rate was based on clinical symptoms, whereas the laboratory-confirmed attack rate used the number of laboratory-confirmed CHIKV cases. The clinical attack rates of specific outbreaks ranged from 0.28% to 73.4% in Indonesia in 2001 and Cambodia in 2012, respectively. The laboratory-confirmed attack rate ranged from 0.13% to 58.3%. Both minimum and maximum laboratory-confirmed attack rates were reported in India in 2010 and 2008, respectively. Other high CHIKV attack rates were 55.6% and 44.7%, reported from Malaysia (2007) and Cambodia (2012) respectively.

### Clinical features of CHIKV infection and atypical manifestations including rare complications

Among the records reporting clinical features associated with CHIKV infection, we only considered the 64 records where all cases described had been laboratory-confirmed (see appendix pp 20–22, Supplementary Table 5). The most commonly reported clinical features were arthralgia, rash, fever, and headache, as they were recorded in at least 50% of studies (Table 1). Chikungunya infection can lead to a broad spectrum of neurological disorders, as well as ocular, cardiovascular, respiratory, skin, auditory, oral, and musculoskeletal manifestations (see appendix pp 23, Supplementary Table 6). Atypical manifestations associated with CHIKV infection begin to be reported only in 2008. There were 47 records on atypical manifestations, of which nearly 38.3% (18 records) were related to neurological disorders (see appendix pp 23, Supplementary Table 6). The second most common was cardiovascular manifestations, at 19.1% (9 records). Additional atypical manifestations were rare dermatologic manifestations, oral candidiasis, hypokalaemic paralysis, and autoimmune polymyositis. Rare complications following CHIKV infection included thrombotic thrombocytopenic purpura, atypical Kawasaki disease, leukemoid reaction, acute auditory neuropathy spectrum disorder, and sudden sensorineural hearing loss with acute respiratory distress syndrome.

**Table 1.** Most commonly reported clinical features of CHIKV infection.

Clinical feature (total number of records = 64)	No. of records	Percentage (%)
Arthralgia	40	62.5
Rash	36	56.3
Fever	35	54.7
Headache	33	51.6
Myalgia	30	46.9
Vomiting	21	32.8
Arthritis	17	26.6
Diarrhoea	14	21.9
Shivering/chills or rigour	13	20.3
Cough	12	18.8
Abdominal pain	10	15.6
Nausea	10	15.6
Conjunctival infection	9	14.1
Lymphadenopathy	8	12.5
Haemorrhage	8	12.5
Sore throat	7	10.9
Eye pain	6	9.4
Pruritis/ itching	6	9.4
Liver involvement	5	7.8
Oral ulcer/gingivitis	5	7.8
Bleeding gums	5	7.8
Back pain	5	7.8
Anorexia	5	7.8

Notes: Percentage = (Number of records reporting the finding/total number of records^64^) × 100. Refer to Supplementary Table 8 for references.

### Death due to CHIKV

There were 12 records, predominantly reported from India and Malaysia, describing 48 cases of death due to (laboratory-confirmed) infection with CHIKV (see appendix pp 24, Supplementary Table 7). Of these cases, 10% were less than 20 years of age, 20% were 20–60 years and 60% were over 60 years of age. India reported the first death due to CHIKV in 1963, and thereafter in 1964, 2006, 2007, and 2011. The most recent reported death attributable to CHIKV infection involved a 12-year old boy from Bangalore, India (2015). Fatalities due to CHIKV infection were also reported from Malaysia in 2008 and 2010 and from Niue in 2015. Fatalities occurred in patients both lacking and with co-morbidities such as hypertension, ischemic heart disease, and diabetes mellitus.

### Vertical transmission

There were 11 records on vertical transmission of CHIKV from mother to neonate during 2008–2016. Nine records were from India and one each from Sri Lanka and Thailand (see appendix pp 25, Supplementary Table 8). The infants showed a spectrum of cutaneous manifestations, however, in some cases, systemic manifestations were also apparent. Mothers were viremic at the time of delivery in all vertically transmitted cases that resulted in serious consequences to the baby.

### CHIKV genotype distribution

**Figure 5.**
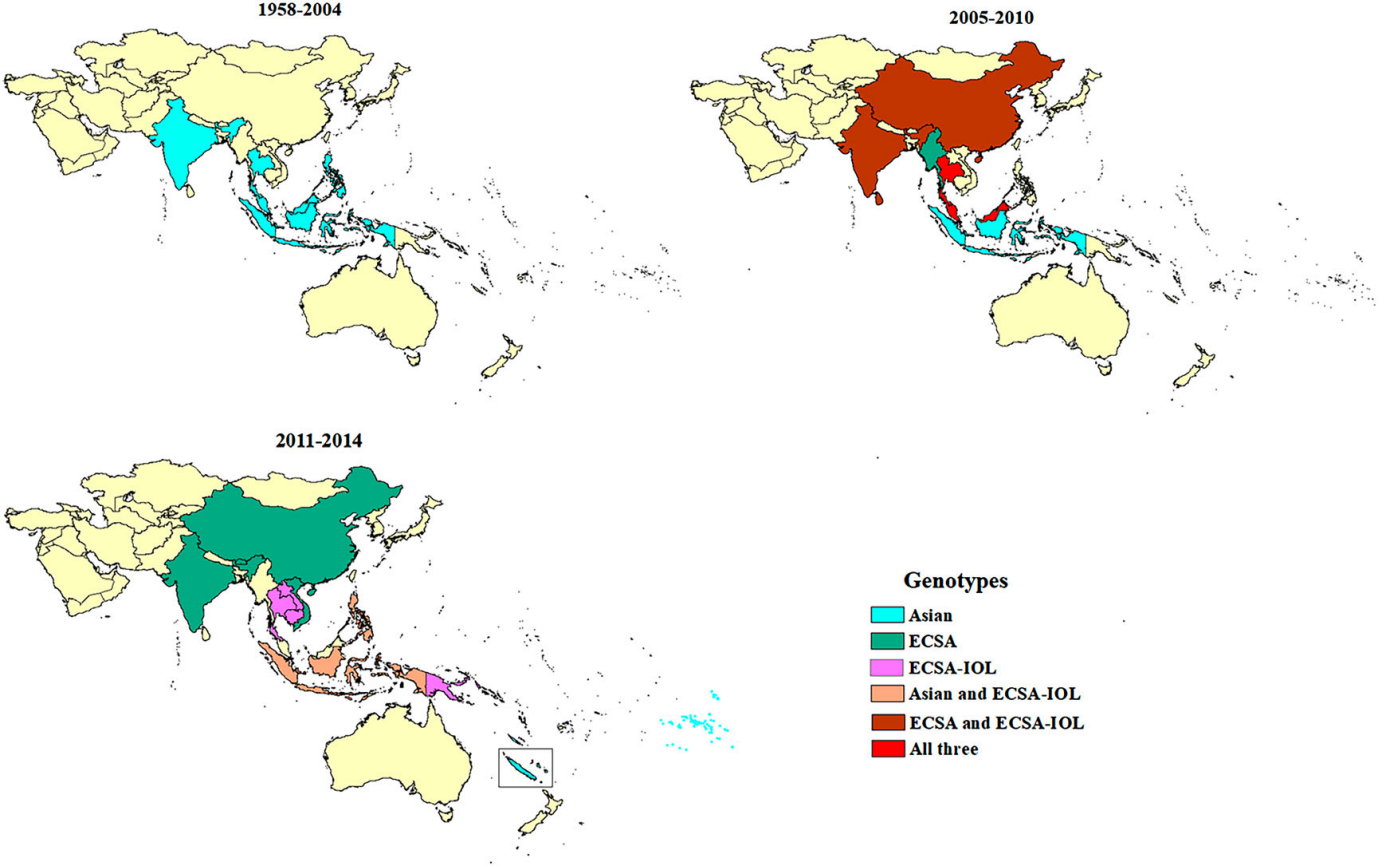
Spatial-temporal distribution of CHIKV genotypes. There was no sequencing information reported for the period 2015–2017 over the period considered for the review.

A total of 95 records reported the genotype of CHIKV circulating in SEA and the WPR (see appendix, pp 26–29, Supplementary Table 9). In the majority (62.1%) of records, CHIKV genotype was determined based on E1 gene sequencing (partial or complete) (see appendix, p 30, Supplementary Table 10). Thailand, India, Indonesia, Malaysia, and Philippines reported sporadic cases and outbreaks of Asian genotype CHIKV during 1950s to 2005, when ECSA strains appear in the region for the first time (Figure 5). Since then, outbreaks involving ECSA strains have occurred almost every year. The first reports of ECSA-IOL strains were made in 2007 from India and Thailand. The majority (80%) of reports concerned strains of ECSA genotype, including 35% of reports documenting IOL strains. Some countries only reported the presence of strains from ECSA genotype (Bhutan, Myanmar, and Vietnam), whereas others only the presence of Asian genotype (New Caledonia and French Polynesia) (Figure 5). Several countries, including Indonesia, Thailand, and Malaysia, experienced circulation of both genotypes. At the time of the review, among countries with only imported cases, Australia and South Korea had reported only ECSA genotype and Japan both genotypes. In India, ECSA-IOL strains were only reported during 2007–2010, while strains reported more recently from 2011–2014 belonged to the ECSA genotype but not to this lineage (see appendix pp 26–29, Supplementary Table 9).

## Discussion

Our findings show that CHIKV has circulated in the Asia-Pacific region since the 1950s, causing sporadic outbreaks in SEA almost every decade from the 1960s. CHIKV activity has steadily expanded since the 1960s, reaching the Western Pacific islands in 2011. Although the first evidence of human CHIKV infection in SEA was obtained from sera collected in India in 1954 [28], the first laboratory-confirmed outbreak occurred in Thailand in the form of an epidemic with dengue co-infection in 1958 [26]. Large CHIKV outbreaks then occurred in SEA between 1961 and 1970 and by 2011, the virus had spread to the countries of Western Pacific islands. Many countries in the SEA region have experienced re-emergence of the disease in recent decades. Unlike a previous report [29], we found intervals between outbreaks in the same country ranged from 6 (Indonesia) to 41 (Sri Lanka) years. It is likely that our review identified earlier reports that had not been previously available. The widely varying intervals observed suggest that improving prediction of CHIKV outbreaks and epidemics will remain challenging.

Our results suggest that India has experienced the highest burden of CHIKV in the Asia-Pacific region. Circulation of CHIKV in India seems to have been almost continuous since the first incursion of the virus into SEA, with the greatest number of reports originating in that country. The highest attack rate for CHIKV in India thus far indicated by WHO is 45% [30], but our data suggests it can reach 58.3% (India, 2008) for laboratory-confirmed cases. Despite the perception that CHIKV-associated mortality was first observed in La Reunion in 2005–2006 [31], we found evidence for fatalities occurring since the first reported outbreak in 1963 in India.

In agreement with previous observations [32], our study confirms that CHIKV causes more severe disease and can be fatal in children and the elderly (>60 years old), even in the absence of co-morbidities, whereas healthy adults usually experience only self-limiting infections. Though the number of fatal cases identified during our review is relatively small, this may be an underestimate given that confirmatory diagnosis is often not performed due to resource limitations [2,33–36] and CHIKV may be mistaken for dengue [1,27,37].

We found that chikungunya can affect all ages but most commonly affects people of 20–60 years old, of either sex. The disease has a broad spectrum of clinical manifestations, with almost all body systems affected. There is a lack of consensus on the common clinical features of CHIKV, as evidenced by different guidelines offered by various health authorities. For example, typical symptoms identified by the WHO Guidelines on Clinical Management of Chikungunya Fever (2008) include fever, arthralgia, back ache, and headache, while the US Centres for Disease Control (CDC) indicate only fever and polyarthralgia [38,39]. Our review failed to identify back pain as a commonly reported feature, with just over 7.8% of reports identifying it as a symptom. Our findings are similar to those of Zim et al*.* [40], who also concluded that arthralgia and rash were commonly associated with CHIKV. While WHO guidelines list rash as an infrequent symptom, we found that rash and headache were reported almost as frequently as fever. According to the aforesaid WHO guidelines, retro-orbital pain, vomiting, and diarrhoea are rarely observed in adults (although occasionally seen in children), yet our results indicate otherwise. An evidence-based consensus on the typical clinical features of chikungunya may aid in improving diagnosis and management of the disease.

Instances of vertical transmission were found to be relatively rare given the large number of CHIKV cases reported during outbreaks in SEA and the WPR. A comprehensive study conducted during the La Reunion outbreak in 2005–2006 identified possible vertical (mother-to-child) transmission of CHIKV from mother to neonate when the mother had viremia at the time of delivery [41]. Similarly, our review identified the instance of vertical transmission of CHIKV from mother to the neonate, with most reports originating from cases in India. CHIKV infection and serious consequences were found in infants who had contracted CHIKV from viremic mothers during delivery, such as neurological or haematological features that can establish permanent disability in the infant [42]. A recent review of CHIKV vertical transmission cases worldwide identified that long-term neurodevelopmental delays occurred in 50% of neonatal infections that showed clinical symptoms [43].

A large proportion of outbreaks in SEA and WPR recorded since 2005 have involved CHIKV strains of the ECSA genotype. Interestingly, in India, we found the predominance of the IOL lineage has declined from a peak during 2007–2011 and may have been displaced by other ECSA strains in recent years. The Asian genotype continues to circulate in SEA and the WPR regions. Following the end date of this systematic review (09/2017), Pyke et al. (2018) reported a case of Asian genotype CHIKV imported into Australia from the Philippines in 2016, indicating recent circulation of the Asian genotype [44]. Press reports from the Philippines during this period indicate a chikungunya outbreak, with over 400 cases [45]. It is clear that the ECSA genotype, including the IOL lineage, has not displaced Asian strains in these regions since 2005, and that both genotypes present an export risk to other areas with suitable vectors.

There are several limitations to this systematic review. First, there is no proper classification or delineation of CHIKV outbreaks and epidemics, possibly due to a lack of consensus on definitions for CHIKV in general [46]. As CHIKV infections can be misdiagnosed/misinterpreted for dengue [27,37,47] virus occurrence and burden of disease in these regions are likely grossly underestimated. A second limitation relates to the quality of information and the frequent absence of laboratory confirmation of cases and outbreaks. Many of the SEA and WPR countries do not have surveillance systems for CHIKV, possibly due to resource constraints. Therefore, under-reporting and small number of studies on CHIKV epidemiology and clinical manifestations are pervasive problems.

Elucidating the epidemiology, circulation and clinical manifestations of CHIKV in the Asia-Pacific is critical to understanding the potential for virus re-emergence. Our findings could improve the accuracy of diagnosis and surveillance, which may assist in the management and prevention of future outbreaks in the region.

## Methods

### Search strategy and selection criteria

The systematic review was performed according to the Cochrane Collaboration guidelines [48], and findings compiled following the “Preferred Reporting Items for Systematic Reviews and Meta-analyses” (PRISMA) format [49]. The term “record” refers to any retrieved document that contains an outcome measure such as epidemiological history, clinical features, and distribution of virus genotypes. The report type, study design, and outcomes reported in each record were systematically screened for inclusion or exclusion in the review, based on predefined eligibility criteria (see appendix pp 31, Supplementary Table 11). All records containing serological surveys, imported cases, sporadic cases, case series, disease clusters, outbreaks and epidemics that reported CHIKV incidence (confirmed by any laboratory method), over any time interval and in human populations were considered for inclusion in this study. Epidemics, outbreaks, and disease clusters were categorized as such based on the classification used by the original authors [46]. CHIKV outbreaks and epidemics were considered eligible for inclusion if at least a subset of cases was laboratory-confirmed. Demography, clinical features, atypical findings including rare complications and vertical transmission were described only using the cases with clear laboratory confirmation. This systematic review covers 47 countries in SEA and the WPR as defined on the 26th of July 2017 (see appendix p 32 for the country list – Supplementary Table 12, and pp 33–38 for full search strategy – Supplementary Table 13). PubMed and Scopus databases, World Health Organisation Western Pacific syndromic surveillance reports were searched by using the keywords: “chikungunya” and “country.” The search was conducted from 26th July 2017 and updated on 17th September 2018. Bibliographies of records identified through these searches were further examined for publications that were not captured in the primary database searches. The bibliographic information was used to extract information from Promed reports (http://www.promedmail.org/) and web databases of the respective governments.

### Data analysis

Records were imported into Endnote X8 bibliographic software (X8.0.1; Thompson Reuters, Philadelphia, USA) and an Excel (Microsoft) file. Duplicate publications were removed, and records containing the same research data/findings published by the same author in different formats/titles were counted only once. Retrieved titles and abstracts, and then potentially eligible full-text records, were screened independently by two reviewers based on the predefined inclusion and exclusion criteria (see appendix p 31 for Supplementary Table 11). Records required an abstract in the English language to be included. Ineligible records were excluded from analyses, and eligible reports were used for data extraction. The following data were extracted and recorded in tables: the type of occurrence (surveillance/serological survey, imported case, case study, sporadic case, case series, disease cluster, outbreak, or epidemic), demography (age and sex), year, and place. Clinical features along with atypical clinical manifestations were only recorded for laboratory-confirmed CHIKV cases. Atypical manifestations were defined as features specific to particular organ systems that were relatively uncommon [42]. Vertical transmission, attack rates and deaths due to CHIKV were reported separately. The viral genotype was recorded according to the year, month, country, and city from where it was reported.

Basic descriptive analysis was performed using GraphPad Prism version 7 (GraphPad Software, La Jolla, California, USA). Maps were produced using ArcGIS version 10.6 (ESRI Inc., Redlands, California, USA). Heat maps to display numbers of records available for each country across the decades were produced using the R package heatmap version 3.3 (R Foundation for Statistical Computing, Vienna, Austria).
